# Red Ginseng Dietary Fiber Shows Prebiotic Potential by Modulating Gut Microbiota in Dogs

**DOI:** 10.1128/spectrum.00949-23

**Published:** 2023-06-27

**Authors:** Hyokeun Song, Junbum Lee, Saehah Yi, Woo-Hyun Kim, Yuna Kim, Beomkwan Namgoong, Ahreum Choe, Gunhee Cho, Jangmi Shin, Youngsik Park, Min Su Kim, Seongbeom Cho

**Affiliations:** a College of Veterinary Medicine and Research Institute for Veterinary Science, Seoul National University, Seoul, South Korea; b Korea Ginseng Corporation, Daejeon, South Korea; c Center for Veterinary Integrated Medicine Research, Seoul National University, Seoul, South Korea; University of Saskatchewan

**Keywords:** dietary fiber, dog, nanopore sequencing, red ginseng, gut microbiota

## Abstract

Red ginseng, widely used in traditional medicine for various conditions, imparts health benefits mainly by modulating the gut microbiota in humans. Given the similarities in gut microbiota between humans and dogs, red ginseng-derived dietary fiber may have prebiotic potential in dogs; however, its effects on the gut microbiota in dogs remain elusive. This double-blinded, longitudinal study investigated the impact of red ginseng dietary fiber on the gut microbiota and host response in dogs. A total of 40 healthy household dogs were randomly assigned to low-dose (*n* = 12), high-dose (*n* = 16), or control (*n* = 12) groups and fed a normal diet supplemented with red ginseng dietary fiber (3 g/5 kg body weight per day, 8 g/5 kg per day, or no supplement, respectively) for 8 weeks. The gut microbiota of the dogs was analyzed at 4 weeks and 8 weeks using 16S rRNA gene sequencing of fecal samples. Alpha diversity was significantly increased at 8 and 4 weeks in the low-dose and high-dose groups, respectively. Moreover, biomarker analysis showed that short-chain fatty acid producers such as *Sarcina* and *Proteiniclasticum* were significantly enriched, while potential pathogens such as *Helicobacter* were significantly decreased, indicating the increased gut health and pathogen resistance by red ginseng dietary fiber. Microbial network analysis showed that the complexity of microbial interactions was increased by both doses, indicating the increased stability of the gut microbiota. These findings suggest that red ginseng-derived dietary fiber could be used as a prebiotic to modulate gut microbiota and improve gut health in dogs.

**IMPORTANCE** The canine gut microbiota is an attractive model for translational studies, as it responds to dietary interventions similarly to those in humans. Investigating the gut microbiota of household dogs that share the environment with humans can produce highly generalizable and reproducible results owing to their representativeness of the general canine population. This double-blind and longitudinal study investigated the impact of dietary fiber derived from red ginseng on the gut microbiota of household dogs. Red ginseng dietary fiber altered the canine gut microbiota by increasing diversity, enriching short-chain fatty acid-producing microbes, decreasing potential pathogens, and increasing the complexity of microbial interactions. These findings indicate that red ginseng-derived dietary fiber may promote canine gut health by modulating gut microbiota, suggesting the possibility of its use as a potential prebiotic.

## INTRODUCTION

Red ginseng is a well-known plant and is widely used in traditional medicine worldwide (1). Red ginseng comprises beneficial components, including ginsenosides and saponins, that improve health by conferring anti-inflammatory, anticancer, and antiobesity properties (2–4). A large quantity of ginseng residue is produced upon extraction of active ingredients from red ginseng, which consists of beneficial compounds, mainly soluble and insoluble dietary fibers, in addition to proteins, amino acids, mineral elements, and other components (5). Furthermore, dietary fiber constitutes approximately one-third of the nutritional composition of red ginseng residues (6). The beneficial effects of dietary fibers in improving metabolic and immune functions have been demonstrated in various studies (7). However, most studies of red ginseng components have focused on the effects of ginseng extract. In contrast, the effects of dietary fibers derived from red ginseng have not been explored fully (8).

The gut microbiota is a complex community of microbes, including bacteria, viruses, archaea, and fungi residing in the host gastrointestinal tract (9). Complex interactions between the host and gut microbiota regulate host fitness, including metabolism, immune response, digestion, and pathogen resistance (10–14). Diverse factors, including host genetics, diet, and environment, shape the gut microbiota (15, 16). Among these, diet is suggested to be the most important driving factor of the gut microbiota (17). As the gut microbiota is closely related to host health, dietary interventions, such as a high-fiber diet, low-sugar diet, and diet supplemented with prebiotics, improve host health by modulating the gut microbiota (18–20). Although a wide range of studies of the response of gut microbiota to dietary intervention have been conducted, the effect of red ginseng dietary fiber on gut microbiota has not been previously studied.

Dogs are companion animals living in close relationships with humans and sharing lifestyles and environments. As dogs are exposed to environmental factors comparable to those experienced by humans that induce lifestyle-related disorders, they frequently suffer from similar diseases and show similar responses to treatment (21). Because of this characteristic that sets them apart from conventional laboratory animals, dogs have been widely used as a sentinel for human health and disorders (22, 23). Moreover, they are frequently exposed to external factors, such as diet and environment, that shape the gut microbiota comparable to that in humans. Therefore, investigating the canine gut microbiota is also important because of its high similarity to that of humans. Compared to other animals, such as laboratory mice and pigs, the canine gut microbiota has the most similar genetic contents to that of humans; it responds to dietary intervention similarly to the human gut microbiota (24). Therefore, improving our understanding of the dynamics of canine gut microbiota in response to dietary interventions is essential, considering the translational value of dogs for human studies.

The present study aimed to reveal the effect of red ginseng dietary fiber on the gut microbiota and host response in dogs to assess its potential as a prebiotic. In the present study, we manufactured red ginseng dietary products for dogs which were composed of soluble and insoluble dietary fibers. To investigate the impact of red ginseng dietary fiber on the gut microbiota of the general canine population, healthy household dogs were selected based on strict exclusion criteria. Longitudinal sampling was conducted at 4-week intervals, and 16S rRNA gene sequencing was used to determine the dose-dependent effects of red ginseng dietary fiber on the gut microbiota of low-dose, high-dose, and control groups. The alterations in taxonomic composition, diversity, and microbial interactions of the gut microbiota were analyzed to investigate the prebiotic potential of red ginseng dietary fiber in modulating gut microbiota and promoting gut health. Moreover, we aimed to identify gut microbial biomarkers that are significantly altered by the intake of red ginseng dietary fiber, which could serve as potential predictors of the health effects of this dietary intervention.

## RESULTS

### General characteristics of participant dogs.

**FIG 1:**
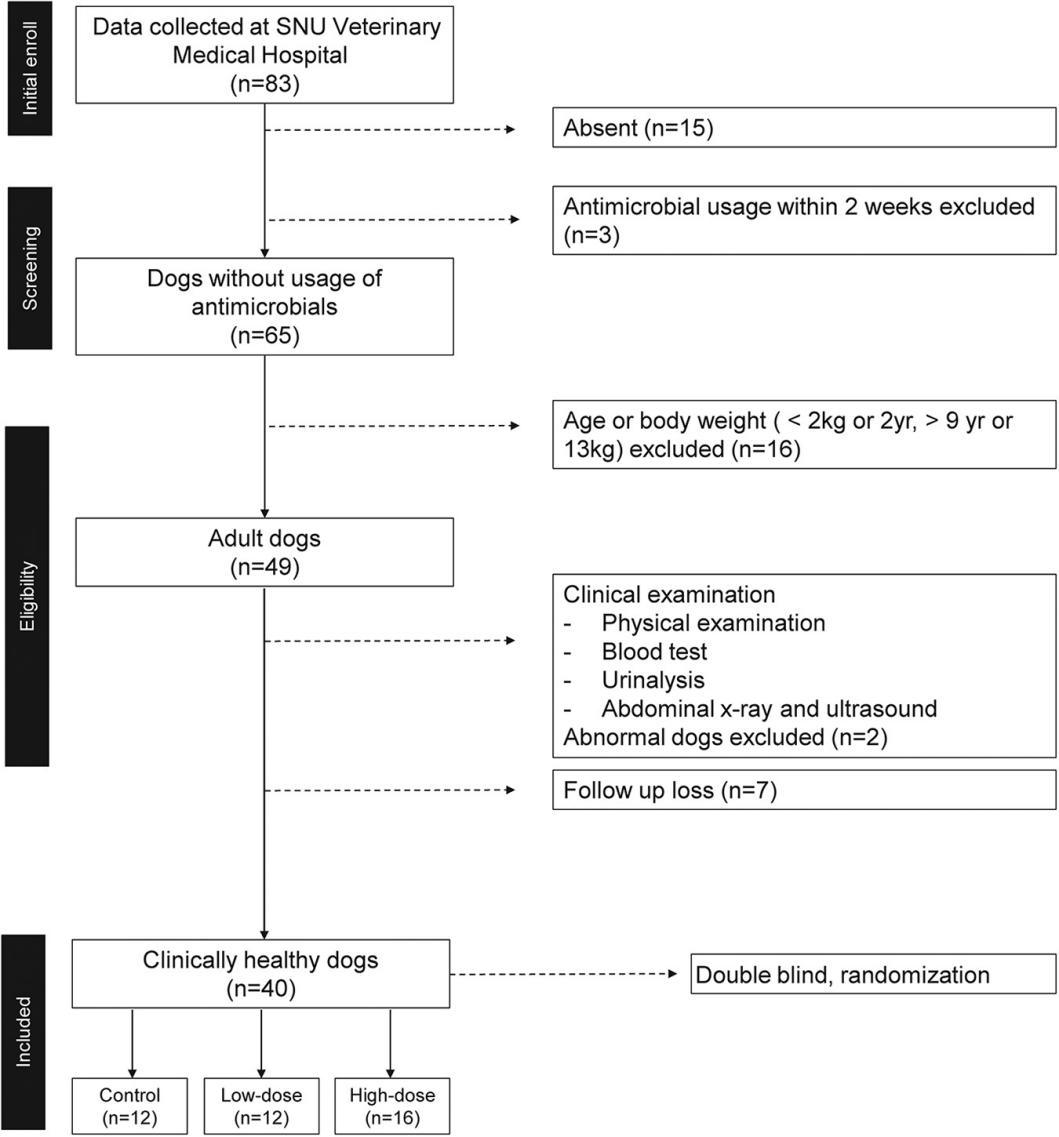
Flow chart demonstrating the exclusion criteria of participant dogs used in the present study.

The exclusion criteria were set to avoid factors such as antimicrobial usage, age, body weight, pregnancy, and abnormalities in health that could affect the gut microbiota and to accurately assess the influence of red ginseng dietary fiber on the gut microbiota. After applying these exclusion criteria, 40 dogs were included in the study out of 83 participant dogs. They were randomly assigned to high-dose intake (8 g/5 kg body weight per day, *n* = 16), low-dose intake (3 g/5 kg per day, *n* = 12), and control (no red ginseng supplement, *n* = 12) groups (Fig. 1).

No significant differences were observed in potential confounding factors, such as age, body weight, sex, and body condition score among the control, low-dose, and high-dose groups (Table 1). Moreover, principal-coordinate analysis (PCoA) based on Bray-Curtis dissimilarity showed no significant differences (*P* > 0.05, permutational multivariate analysis of variance [PERMANOVA]) in gut microbiota according to these factors (see Table S1 in the supplemental material). All participant dogs showed insignificant changes in their clinical status during the study period and were clinically healthy according to physical examination and laboratory analysis (Table S2). No owner reported noticeable side effects or clinical signs during the intake of red ginseng dietary fibers.

**TABLE 1:** Demographics of participant dogs

Variable^*a*^	Control (*n* = 12)	Low-dose (*n* = 12)	High-dose (*n* = 16)	*P* value^*c*^
Age (yrs)	4.75 ± 2.26	5.83 ± 1.59	5.31 ± 2.21	0.8914
Body wt (kg)	6.37 ± 3.44	5.87 ± 3.00	6.35 ± 3.15	0.4447
Body condition score^*b*^	5.00 ± 0.86	5.38 ± 1.02	5.33 ± 0.65	0.5005
Sex (*n*)				0.07^*d*^
Male	3	6	11	
Female	9	6	5	

a Age, body weight and body condition score values are the mean ± standard deviation.

b Score that demonstrates relative dog fatness, as evaluated by veterinarian’s physical examination.

c For age, body weight and body condition score, the *P* value was evaluated using an ANOVA test.

d The *P* value was evaluated using a Chi-square test.

### Taxonomic composition of the canine gut microbiota.

**FIG 2:**
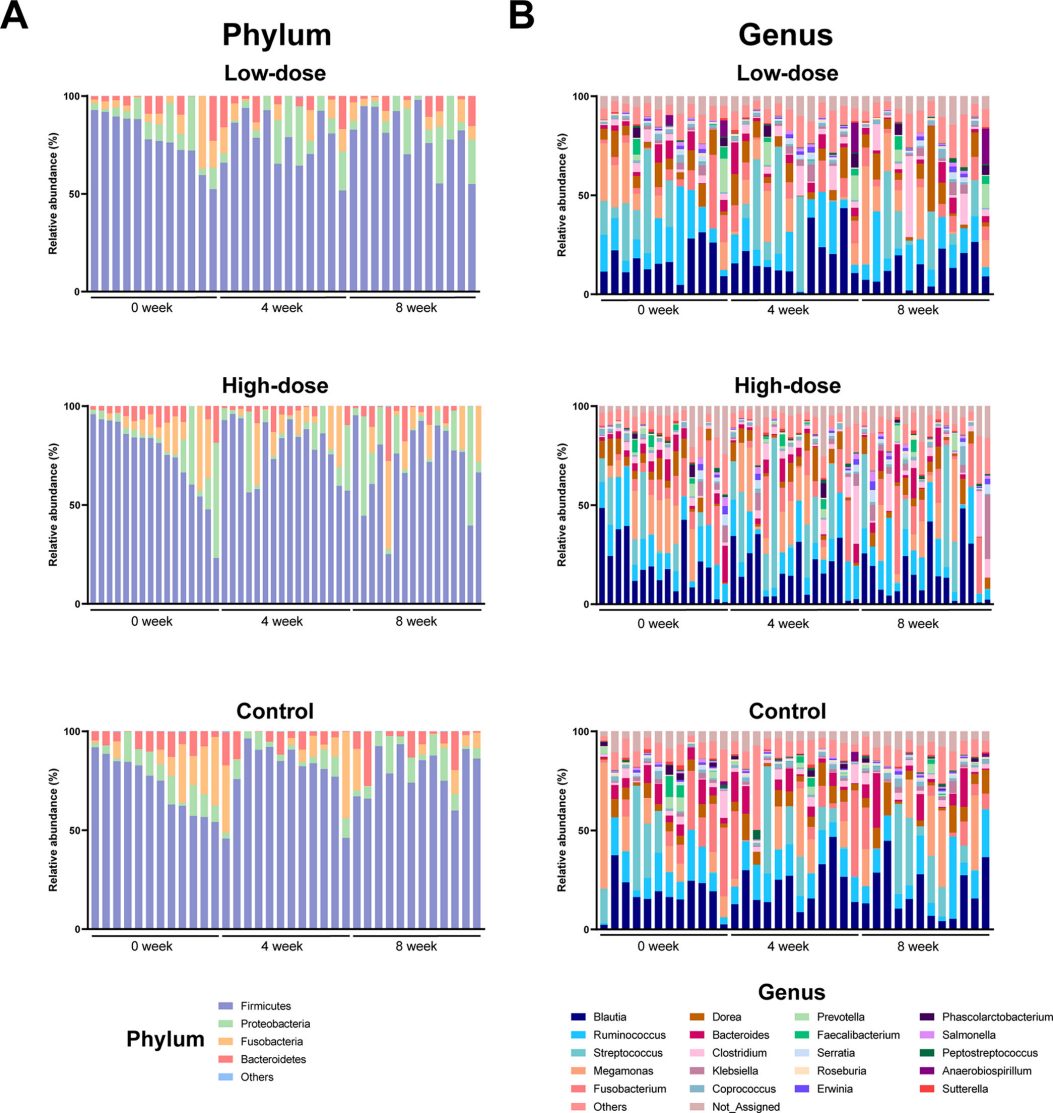
(A and B) Taxonomic composition of the canine gut microbiota during the intake of red ginseng dietary fiber at the (A) phylum and (B) genus levels. Only the top 20 genera are shown.

To assess the effects of red ginseng dietary fiber, we first analyzed the alteration in the taxonomic composition of the gut microbiota at the phylum and genus levels (Fig. 2). Before the red ginseng dietary fiber intake (0 weeks, baseline), *Firmicutes*, *Proteobacteria*, *Bacteroidetes*, and *Fusobacteria* were the most dominant phyla in the control, low-dose, and high-dose groups (four-taxa combined averages of 99.90%, 99.97%, and 99.98%, respectively). The dominance of these taxa in the three groups was maintained after 4 (four-taxa combined averages of 99.90%, 99.92%, and 99.93%, respectively) and 8 weeks (four-taxa combined averages of 99.90%, 99.95%, and 99.92%, respectively) of red ginseng dietary fiber intake.

At the genus level, *Blautia* and *Ruminococcus* were the dominant genera in the control, low-dose, and high-dose groups at 0 (*Blautia*, average of 17.64%, 18.36%, and 19.71%, respectively; *Ruminococcus*, average of 16.23%, 14.13%, and 11.49%, respectively), 4 (*Blautia*, average of 21.04%, 18.65%, and 18.20%, respectively; *Ruminococcus*, average of 13.78%, 13.11%, and 12.23%, respectively), and 8 weeks (*Blautia*, average of 18.53%, 14.41%, and 19.24%; *Ruminococcus*, average of 12.24%, 12.93%, and 13.65%, respectively) of red ginseng dietary fiber intake.

### Shift of the diversity of the canine gut microbiota.

**FIG 3:**
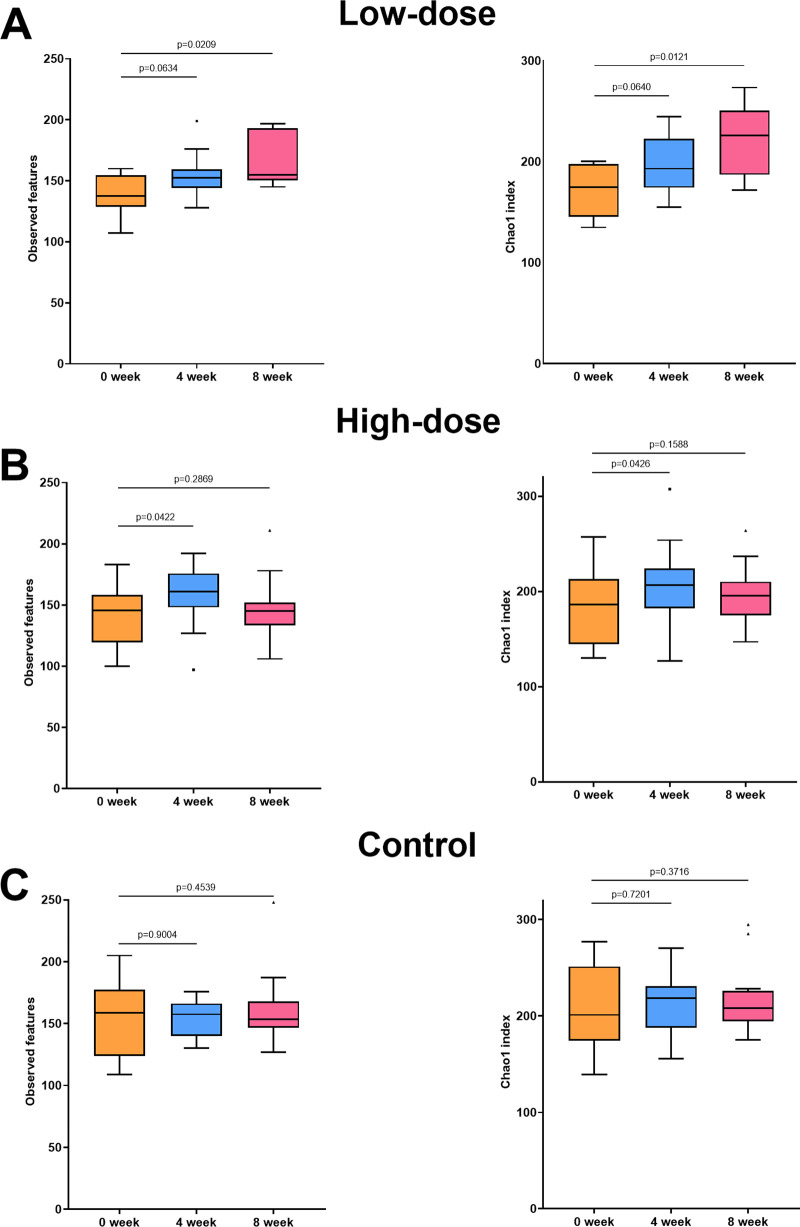
Alteration in alpha diversity of the gut microbiota of dogs during intake of red ginseng dietary fiber. (A to C) Box plots show the alteration of the number of observed features and Chao1 index in the (A) low-dose, (B) high-dose, and (C) control groups. The boxes represent the interquartile range of the data. The line inside the boxes represents the median of the data. The whiskers extending from the boxes represent the range of the data, excluding any outliers, with the whisker length defined as 1.5 times the interquartile range. Any data points beyond the whiskers are plotted as individual points and are considered outliers.

We analyzed the alteration of the diversity of the gut microbiota based on the number of observed species and the Chao1 index. As shown in Fig. 3A, both indices of the alpha diversity were significantly increased (Wilcoxon test, *P* < 0.05) after 8 weeks in the low-dose group. However, no significant difference was observed at 0 and 4 weeks (*P* > 0.05). As shown in Fig. 3B, both indices of the alpha diversity increased after 4 weeks in the high-dose group (*P < *0.05), while no significant difference was observed at 0 and 8 weeks in the high-dose group (*P > *0.05). In the control group, no significant alteration (*P* > 0.05) of alpha diversity was observed at any time point (Fig. 3C). No significant alteration (*P* > 0.05) of beta diversity was observed at any time point in all groups (Fig. S1).

### Differential abundance analysis of the canine gut microbiota.

**FIG 4:**
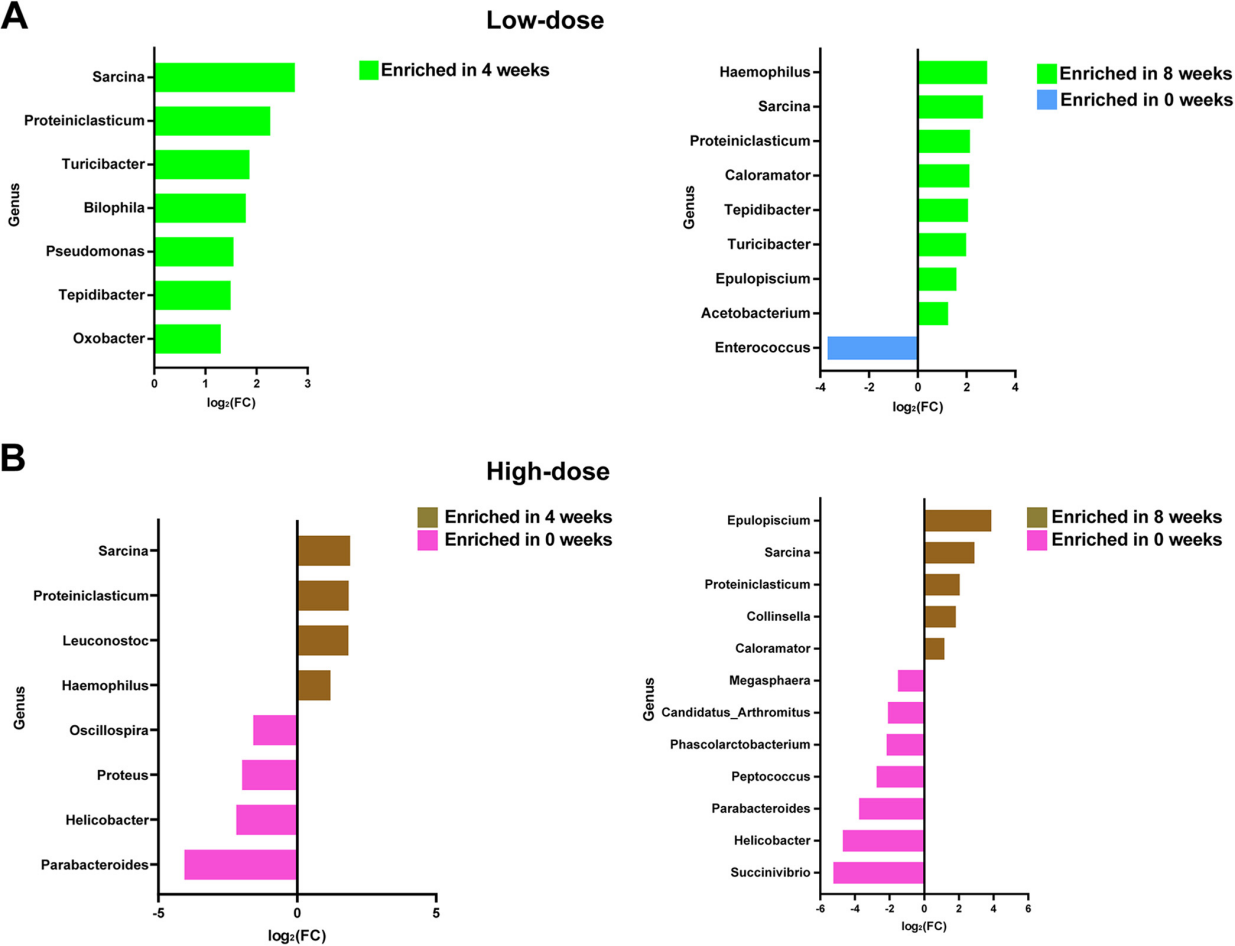
Differential abundance analysis of the canine gut microbiota. (A) Results of edgeR analysis between 0 and 4 weeks and 0 and 8 weeks in the low-dose group. (B) Results of edgeR analysis between 0 and 4 weeks and 0 and 8 weeks in the high-dose group.

We used edgeR (Fig. 4) to analyze specific gut microbiota components, which differed significantly by red ginseng dietary fiber intake. In the low-dose group, *Sarcina*, *Proteiniclasticum*, *Turicibacter*, *Bilophila*, Pseudomonas, *Tepidibacter*, and *Oxabacter* were significantly enriched (*P* < 0.05) after 4 weeks, whereas Haemophilus, *Sarcina*, *Proteinclasticum*, *Caloramator*, *Tepidibacter*, *Turicibacter*, *Epulopiscium*, and *Acetobacterium* were significantly enriched (*P* < 0.05), and *Enterococcus* was significantly decreased (*P* < 0.05) after 8 weeks. In the high-dose group, *Sarcina*, *Proteinclasticum*, *Leuconostoc*, and Haemophilus were significantly enriched (*P* < 0.05) and *Parabacteroides*, *Helicobacter*, Proteus, and *Oscillospira* were significantly decreased (*P* < 0.05) after 4 weeks, and *Epulopiscium*, *Sarcina*, *Proteiniclasticum*, *Collinesella*, and *Caloramator* were significantly enriched (*P* < 0.05), and *Succinivibrio*, *Helicobacter*, *Parabacteroides*, *Peptococcus*, *Phascolarctobacterium*, “*Candidatus* Arthromitus,” and *Megasphaera* were significantly decreased (*P* < 0.05) after 8 weeks.

### Complexity of microbial interactions in the canine gut microbiota.

**FIG 5:**
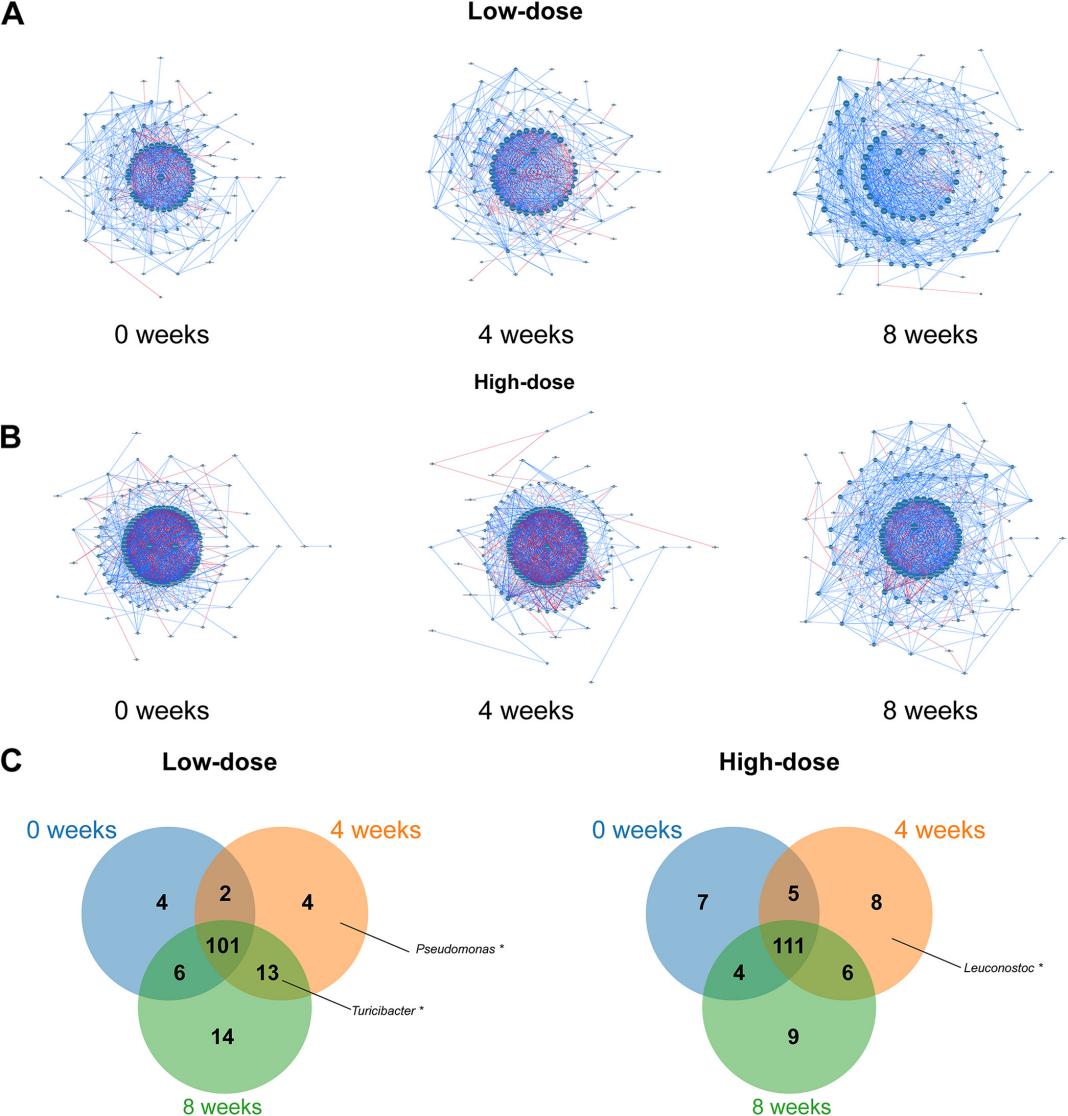
Alterations in the ecological network of the canine gut microbiota owing to the intake of red ginseng dietary fiber. (A and B) Cooccurrence network of the gut microbiome in the (A) low-dose and (B) high-dose groups at the genus level. The cooccurrence network was generated using NAMAP with Spearman’s correlation. Interactions showing *P < *0.05 and *r *> 0.7 were considered significant. Positive and negative correlations are shown as blue and red lines, respectively. (C and D) Venn diagrams show the shared and unique nodes of the cooccurrence networks in the (C) low-dose and (D) high-dose groups. Asterisks denote the genus that was enriched after 4 weeks or 8 weeks in differential abundance analysis.

To elucidate the shift in ecological interactions between gut microbes according to the intake of red ginseng dietary fiber, we constructed cooccurrence networks for the time points in the high-dose and low-dose groups. In the low-dose group, 113 genera (nodes) and 768 correlations (edges) were significant (*P < *0.05, *r *> 0.7) at 0 weeks. After 4 weeks of intake of red ginseng dietary fiber, 120 nodes and 920 edges were observed, and after 8 weeks, 134 nodes and 879 edges were observed (Fig. 5A). The network of canine gut microbiota in the low-dose group shared 101 nodes among time points. The numbers of unique nodes and edges were 4, 4, and 14 at 0, 4, and 8 weeks, respectively. In the high-dose group, 127 nodes and 1,213 edges were significant (*P < *0.05, *r* > 0.7) at 0 weeks. After 4 weeks, 130 nodes and 1,077 edges, and after 8 weeks, 130 nodes and 1,025 edges were observed (Fig. 5B). The network of canine gut microbiota in the high-dose group shared 111 nodes among time points. The numbers of unique nodes were 7 at 0 weeks, 8 after 4 weeks, and 9 after 8 weeks.

## DISCUSSION

The canine gut microbiota is attracting attention for its translational value, as it has highly similar genetic contents and responds similarly to dietary interventions as those in humans (24). While previous studies have mainly focused on the effect of red ginseng extract or products on the gut microbiota, the effect of red ginseng dietary fibers has not been studied. Moreover, most *in vivo* studies of the effects of red ginseng compounds on the gut microbiota have been conducted using animals in strictly controlled environments, such as laboratory mice. In contrast, studies of dogs that share an environment with humans are scarce. In the present study, we investigated the effect of red ginseng dietary fiber on the gut microbiota and host response in dogs. Healthy household dogs were selected through strict exclusion criteria to represent the effect of red ginseng dietary fiber on the gut microbiota in the general canine population. The present study controlled for potential variable factors, such as sex and body weight, that may affect the microbial composition by conducting a longitudinal follow-up study. The present study is noteworthy, as it demonstrates the prebiotic potential of the red ginseng diet by modulating the gut microbiota.

The findings reveal significant increases in alpha diversity in both the low-dose and high-dose groups, with the low-dose group showing a significant increase after 8 weeks and the high-dose group showing a significant increase after 4 weeks. These results demonstrate that the effect of red ginseng dietary fiber on the diversity of gut microbiota may occur in a dose-dependent manner. Consistently, a previous study of the three types of dietary fibers showed a dose-dependent effect on the diversity of gut microbiota (25). Moreover, our results showed that the diversity of gut microbiota increased in the high-dose group after 4 weeks and decreased to comparable baseline levels at 8 weeks. This may be due to the resilience of gut microbiota, which is a homeostatic characteristic of the microbiota to revert to its original state (26). As dietary change is an external factor that alters the gut microbiota, an initial increase in diversity may have occurred in the high-dose group. In addition, increased diversity of the gut microbiota can positively affect host health by maintaining gut homeostasis (27), whereas decreased diversity is associated with various gastrointestinal, metabolic, and immune diseases, indicating dysbiosis of the gut microbiota (28, 29). Thus, red ginseng dietary fiber may improve host health by increasing gut microbial diversity.

We further investigated the altered specific genera, which are putative biomarker microbes of the gut microbiota, by red ginseng dietary fiber intake. Differential abundance analysis showed two key short-chain fatty acid (SCFA) producers among the microbes that had significant changes at different time points, *Sarcina* and *Proteiniclasticum*, which were significantly increased in both the low-dose or high-dose groups after 4 and 8 weeks. These microbes produce SCFAs by fermenting nondigestible carbohydrates (30, 31), which play a major role in host health. Dietary modification, particularly a fiber-rich diet, is a major factor associated with increased SCFA producers in the gut (32). As the red ginseng material used in this study is rich in various dietary fibers, they may have promoted significant growth of SCFA producers, which is consistent with previous studies (33, 34). Collectively, our results demonstrate that red ginseng dietary fiber intake may positively affect host health by enriching SCFA producers in the gut microbiota.

Our differential abundance analysis showed that microbes significantly decreased after red ginseng dietary fiber intake were mostly potential pathogens, including *Helicobacter*, *Peptococcus*, and Proteus, indicating that red ginseng dietary fiber intake confers resistance to colonization of these pathogens. This may be due to an increase in microbial diversity or an increase in SCFA producers in the gut microbiota. Increased richness of microbiota can confer resistance to pathogen colonization (35), which is consistent with our results. SCFAs produced by the gut microbiota suppress the growth of pathogens *in vitro* and *in vivo* (36–38). Considering that *Helicobacter* is a life-threatening pathogen in both humans and dogs and is often transmitted from dogs to owners (39), decreased *Helicobacter* in the gut microbiota of dogs after intake of red ginseng dietary fiber may improve dog health and, subsequently, human health.

As complicated interactions between gut microbes regulate the stability of gut microbiota (40, 41), we investigated the alteration of the microbial network by the intake of red ginseng dietary fiber. We observed that distinct alterations in network structures occurred because of the intake of red ginseng dietary fiber. The number of nodes, which demonstrates the complexity of microbial interactions (42), increased after the intake of red ginseng dietary fiber in both the low-dose and high-dose groups. This may be because red ginseng dietary fiber intake increased the richness of the gut microbiota, which is consistent with our diversity analysis. In the high-dose group, the number of nodes increased after 8 weeks, although gut microbiota diversity did not significantly increase. This may be because the high-dose treatment induced changes in the functional roles of the different microbial species, which are not reflected in the richness measures. For instance, certain gut microbes may be changing their metabolic activity or interactions with other microbes, which could increase the complexity of the gut microbiota network. Furthermore, the unique nodes observed after red ginseng dietary fiber intake were identified as mainly SCFA producers, including *Turicibacter* and *Leuconostoc*. This is consistent with our differential abundance analysis showing significant enrichment in SCFA producers after red ginseng dietary fiber intake in both treated groups. Increased complexity of the microbial network may improve canine health, as SCFA producer-mediated microbial interactions are associated with host health factors such as immunity and metabolism (43).

In contrast to laboratory dogs maintained in a controlled environment (44), participant dogs enrolled in this study were household dogs that share the environment with their owners. Laboratory dogs may have less variability in gut microbiota composition since they have similar host and environmental factors that affect the gut microbiota. However, aspects that make laboratory animals less variable may also produce less generalizable results to represent the overall population of companion dogs that comprise different sexes, breeds, life stages, body weights, and body conditions (45). The fact that the present study was conducted in household dogs, as opposed to laboratory dogs, increases the generalizability of the results, as the dogs in the present study are representative of the population of dogs in the general household environment. Furthermore, we conducted a longitudinal follow-up study and pairwise analysis of change in the gut microbiota diversity among time points to compensate for the putative limitations of household dogs. In addition, strict exclusion criteria and randomization were applied for participant dogs to minimize the factors that may affect the gut microbiota, resulting in the exclusion of half of the initially recruited dogs from the study. As a result, these factors were not identified as confounding in the present study, as no significant variation in the gut microbiota induced by these factors was observed at baseline. Therefore, the present study may provide a robust representation of the canine population and a reproducible understanding of the dynamics of the gut microbiota in response to the intake of red ginseng dietary fiber for further studies.

The putative limitation of the present study is that only small-breed dogs were included to control for confounding factors, which may limit the generalizability of the results to larger dog breeds. Additionally, the study only analyzed three time points (baseline, 4 weeks, and 8 weeks), which may not fully capture the complexity of the dynamics in the gut microbiota over time. Future studies with larger and more diverse samples, as well as more frequent and long-term sampling, may provide a more comprehensive understanding of the effects of dietary fiber on the gut microbiota in dogs.

All participating dogs enrolled in this study were clinically healthy before red ginseng dietary fiber intake and showed no abnormal signs during the study period. Therefore, red ginseng dietary fiber intake did not affect dog health negatively. As our study revealed the prebiotic potential of red ginseng dietary fiber in healthy dogs, further studies exploring the effect of red ginseng dietary fiber in dogs with abnormal states, such as dysbiosis of the gut microbiota or disease, may improve our knowledge of the clinical effect of red ginseng dietary fiber and the possibility of red ginseng dietary fiber for potential pharmabiotics.

### Conclusions.

The present double-blind, longitudinal study revealed alterations in the canine gut microbiota owing to long-term intake of dietary fiber derived from red ginseng. Intake of red ginseng dietary fiber increased the diversity of gut microbiota in a dose-dependent manner, increased the abundance of SCFA producers and complexity of microbial interactions, and decreased the abundance of potential pathogens. These results demonstrate that red ginseng dietary fiber may promote canine gut health by modulating gut microbiota, suggesting the possibility of its use as a potential prebiotic for dogs and humans.

## MATERIALS AND METHODS

### Ginseng materials.

Red ginseng feed was processed using water-soluble ingredients extracted from 6-year-old Korean ginseng. During processing, pulverization was carried out to increase swelling during molding and then was formulated through extrusion. The formulated product was dried and coated with palatability enhancer liquid and powder under vacuum. Details of the compounds in red ginseng dietary fiber used in this study are shown in Table S3.

### Study design and sample collection.

This study was reviewed and approved by the Institutional Animal Care and Use Committee of Seoul National University (SNU-210115-2). A total of 83 clinically healthy and privately owned dogs were enrolled in the present study, and written consent was obtained from the owners after thoroughly explaining the study at the Seoul National University Veterinary Medical Hospital. The following exclusion criteria were set: (i) use of antibiotics in the last 2 weeks, (ii) dogs less than 2 years of age or older than 9 years of age, (iii) small-breed dogs more than 13 kg or less than 2 kg of body weight, (iv) pregnant dogs, (v) abnormalities in health screening, including physical examination, blood analysis, urinalysis, and abdominal radiology ultrasonography. The owners were told to feed the dogs the provided red ginseng dietary fiber daily, in addition to their original regular diet (low protein, high carbohydrates), for 8 weeks. After every 4 weeks, the health status of the participant dogs was examined by experienced veterinarians. The assessment included history, physical examination, and blood collection for a complete blood count and biochemical profile. Fecal samples were collected monthly and transferred to the laboratory for metagenomic analysis.

### Fecal DNA extraction, library preparation, and 16S rRNA gene full-length nanopore sequencing.

DNA was extracted from fecal samples of dogs using a Qiagen Power Fecal DNA Pro kit. From 10 ng of extracted DNA, full-length bacterial 16S gene rRNA amplicons were generated by PCR using barcode primers provided in the 16S Barcoding Kit. The amplicons from each sample were purified and cleaned using AMPure XP beads. Purified amplicons were pooled at equivalent concentrations. For a single sequencing, a total of 24 samples were pooled. Pooled barcoded libraries were loaded into flow cells (version R9.4), and long-read 16S rRNA sequencing was conducted using a MinION platform. The average sequencing depth was 32,525.98 reads per sample.

### Bioinformatics and statistical analysis.

Statistical analyses were used to reduce the impact of confounding variables, such as age, body weight, sex, and body condition score between the three participant dog groups, control, low-dose, and high-dose. One-way analysis of variance (ANOVA) was used to analyze continuous variables (age, body weight, and body condition score), and the Chi-square test was used to analyze categorical variables (sex) for statistical significance.

Quality control of the raw sequence data was conducted by trimming barcode sequences and discarding reads with lengths below 1,400 bp. Preprocessed sequence data were base-called using MinKNOW with the superaccuracy base-calling option. The taxonomy profile and feature table of sequence data were generated using Kraken 2 (46) using the Greengenes database (version 13.5).

Downstream analysis was performed using R packages (47, 48). The alpha diversity of the gut bacteria was evaluated using the number of observed features and the Chao1 index. A paired test was used to evaluate the significance of differences in the alpha diversity. Beta diversity of the canine gut microbiota was evaluated based on Bray-Curtis dissimilarity. Differential abundance analysis of gut microbiota was performed using edgeR (49). The ecological network of gut microbiota was constructed with network analysis for metagenomic abundance profiles (NAMAP) based on Spearman’s correlations (50) with *r *> 0.7 and *P < *0.05 as the cutoff for significance.

### Data availability.

The sequence data generated in this study were deposited in the National Center for Biotechnology Information Short Read Archive database under accession number PRJNA868512.
