# Supplementary material for: Red Ginseng Dietary Fiber Shows Prebiotic Potential by Modulating Gut Microbiota in Dogs
Source: Microbiol Spectr. 2023 Jun 27;11(4):e00949-23. doi: 10.1128/spectrum.00949-23 (PMC10433987; doi:10.1128/spectrum.00949-23)
Supplement: Supplemental file 1 — Supplemental material. Download spectrum.00949-23-s0001.docx, DOCX file, 0.3 MB [file spectrum.00949-23-s0001.docx]

**Supplementary Table 1. Beta diversity of gut microbiota of participant dogs at 0 week**

| **Category** | **R^2^** | **P value^a^** |
| --- | --- | --- |
| Group | 0.021905 | 0.978 |
| Sex | 0.0094332 | 0.922 |
| Body condition score | 0.096604 | 0.549 |
| Age | 0.20356 | 0.236 |

^a^ Evaluated by PERMANOVA test

**Supplementary Table 2. Complete blood count, blood chemistry and body weight analysis of participant dogs during the study period.**

Submitted as supplementary file.

**Supplementary Table 3. Detailed components of ginseng materials used in the study.**

| **Components** | **Concentration (mg/g)** |
| --- | --- |
| Polysaccharide | 25.44 |
| Arginine-fructose-glucose | 3.58 |
| Ginsenosides^a^ | 37.80 |
| Rg1 | 0.40 |
| Re | 0.14 |
| Rf | 0.43 |
| Rh1 | 0.65 |
| Rg2s | 0.34 |
| Rb1 | 1.60 |
| Rc | 0.44 |
| Rb2 | 0.43 |
| Rd | 0.17 |
| Rg3s | 1.99 |
| Rg3r | 1.22 |
| Saponin | 63.18 |

^a^ mg/kg

**Supplementary Table 4. Nodes of the ecological network of gut microbiome in the low-dose and high-dose groups.**

Submitted as supplementary file

**Supplementary Figure 1.** Principal coordinates analysis of gut microbiota of dogs based on Bray-Curtis dissimilarity.

**
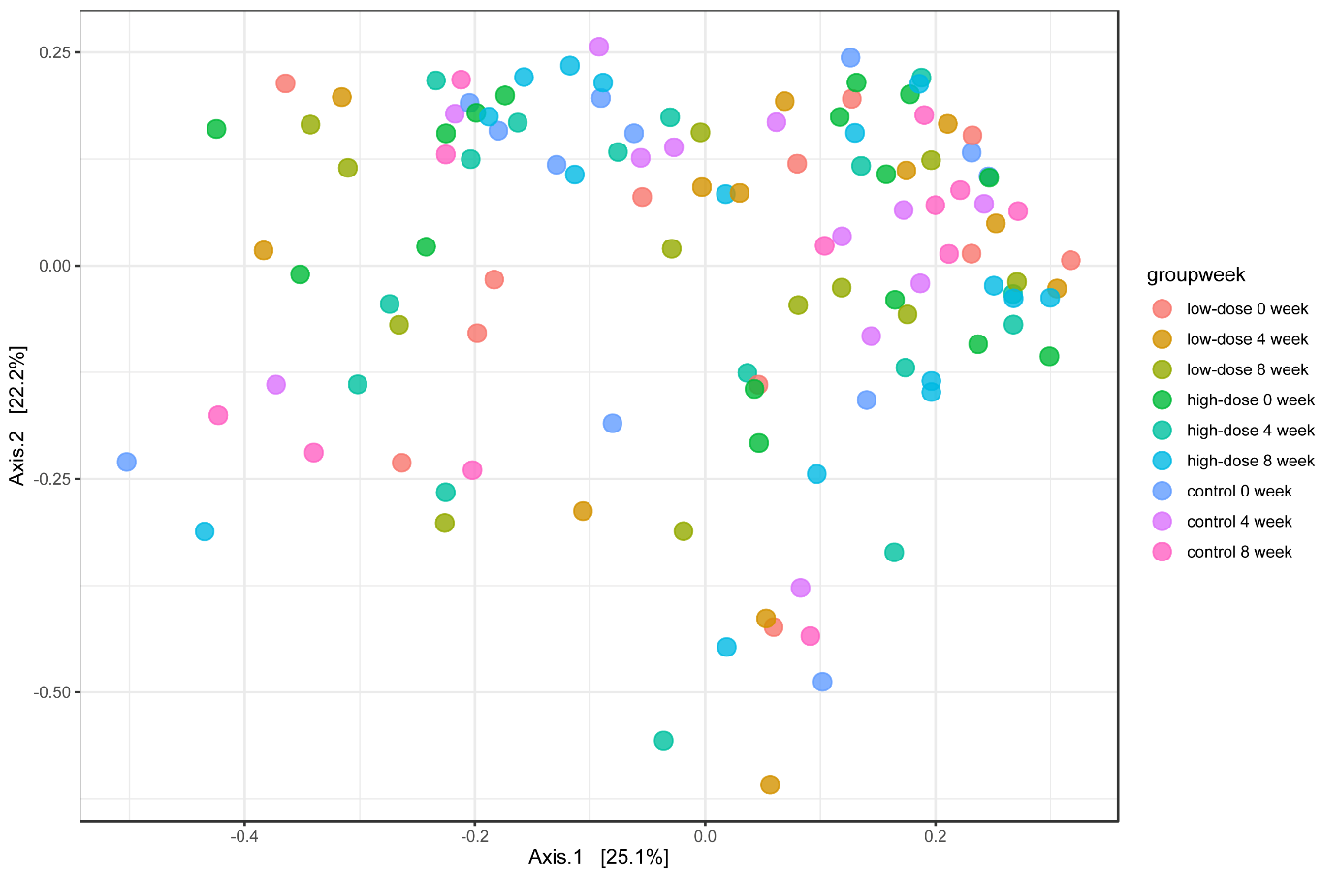
**
